# Did the COVID-19 pandemic delay treatment for localized breast cancer patients? A multicenter study

**DOI:** 10.1371/journal.pone.0304556

**Published:** 2024-05-31

**Authors:** Ke Zhou, Marie Robert, Valérie Seegers, Audrey Blanc-Lapierre, Stéphane Savouroux, Frédéric Bigot, Jean-Sébastien Frenel, Mario Campone, Thierry Conroy, Frédérique Penault-Llorca, Jean-Luc Raoul, Martine M. Bellanger

**Affiliations:** 1 Department of Human and Social Sciences, Institut de Cancérologie de l’Ouest (ICO), Saint-Herblain, France; 2 Department of Medical Oncology, Institut de Cancérologie de l’Ouest, Saint-Herblain, France; 3 Department of Biostatistics, Institut de Cancérologie de l’Ouest, St-Herblain, France; 4 Department of Health Promotion and Prevention, Institut de Cancérologie de l’Ouest (ICO), Saint-Herblain, France; 5 Department of Medical Oncology, Institut de Cancérologie de l’Ouest, Angers, France; 6 Department of Medical Oncology, Institut de Cancérologie de Lorraine, Vandoeuvre-lès-Nancy, France; 7 Department of Biopathology and INSERM U1240, Centre Jean Perrin, Clermont-Ferrand, France; Local Health Authority Caserta: Azienda Sanitaria Locale Caserta, ITALY

## Abstract

**Background:**

Longer times between diagnosis and treatments of cancer patients have been estimated as effects of the COVID-19 pandemic. However, relatively few studies attempted to estimate actual delay to treatment at the patient level.

**Objective:**

To assess changes in delays to first treatment and surgery among newly diagnosed patients with localized breast cancer (BC) during the COVID-19 pandemic.

**Methods:**

We used data from the PAPESCO-19 multicenter cohort study, which included patients from 4 French comprehensive cancer centers. We measured the delay to first treatment as the number of days between diagnosis and the first treatment regardless of whether this was neoadjuvant chemotherapy or surgery. COVID-19 pandemic exposure was estimated with a composite index that considered both the severity of the pandemic and the level of lockdown restrictions. We ran generalized linear models with a log link function and a gamma distribution to model the association between delay and the pandemic.

**Results:**

Of the 187 patients included in the analysis, the median delay to first treatment was 42 (IQR:32–54) days for patients diagnosed before and after the start of the 1^st^ lockdown (N = 99 and 88, respectively). After adjusting for age and centers of inclusion, a higher composite pandemic index (> = 50 V.S. <50) had only a small, non-significant effect on times to treatment. Longer delays were associated with factors other than the COVID-19 pandemic.

**Conclusion:**

We found evidence of no direct impact of the pandemic on the actual delay to treatment among patients with localized BC.

## Introduction

Delays in the diagnosis and treatment of patients with cancers can have adverse effects on clinical outcomes (e.g. overall or disease-free survivals) and quality of life [1–3]. A systematic review by Hanna and colleagues showed an associated increase in the risk of death with a four-week delay in treatment of several cancer types including bladder, colorectal, breast, cervical, and head and neck cancers [2]. However there is a clear need for a standardized methodological approach, including lag time definitions [3]. Delays in diagnosis and treatment were already a global public health issue and an important matter for individual patients prior to the COVID-19 pandemic [4]. The pandemic has exacerbated this problem, as several changes in health-care delivery occurred as a result of efforts to control outbreaks, along with patients’ fears leading to delays in seeking medical care.

The real-world impacts of the pandemic occurred at different stages of the cancer care continuum. The delay from symptom onset to diagnosis increased due to both patients’ worries and the lockdown and thus, limited access to GPs. Furthermore, hospitals witnessed heavy workload and hence increased the lag time between diagnosis and medical care. This increase in time, whether due to COVID-related precautions or the load of COVID patients itself, led to delayed surgeries in many countries because of a restricted number of beds available and overburdened clinicians. The increase in lag time to a cancer diagnosis resulted in more advanced tumor stages at presentation during the late-pandemic period compared to the pre-pandemic period [5–8]. Delays in cancer diagnosis were associated with expected increased deaths, up to five years after diagnosis, as reported by a UK population-based modelling study [6]. Longer time intervals between diagnosis and treatments are expected, which affect the survival of cancer patients [9], particularly if these delays are as long as 3 to 6 months [10]. However, the magnitude of the effects depend strongly on the type of cancer and the lag time intervals measured in the cancer care continuum [3, 10]. To some extent, planned cancer surgery was sensitive to the stringency of the pandemic lockdown (i.e., light or moderate restrictions and full lockdowns), as reported in different income country group levels [11].

Regarding breast cancer (BC), which is the focus of our study presented below, essential surgical (i.e., oncologic surgery) and medical oncology were not suspended. Cancer centers adjusted treatment type and regimen during the COVID-19 period and performed more breast conserving surgery and simple mastectomies, as well as more neoadjuvant therapy [5, 12], and used hypofractionation radiotherapy to reduce admission frequency [5]. This was part of the official French guidelines to protect cancer patients against SARS-CoV-2 infection, along with adjusted dosing schedules of chemotherapy and access to telemedicine (i.e., remote consultations via telephone or videoconference) [13, 14]. Based on nationwide trends from 2010 to 2021 for oncologic surgery, BC was found to be the least affected cancer during the pandemic, in terms of case volume [15]. However, longer times than in standard-of-care occurred within that period. The extent of these delays is crucial, as survival outcomes in early-stage breast cancer are affected by the length of the interval between diagnosis and surgery which helped set recommendations to prioritize care of some BC patients after triage during the COVID-19 pandemic [16, 17].

In France, several series have estimated the impact of pandemic-related diagnosis delays within the UNICANCER Comprehensive Cancer Center network and their effects on patients with newly diagnosed cancer, including BC [18]. Additionally, in the largest French cancer centers, the impacts of treatment delays on BC patients’ outcomes were predicted using simulation models. Delays were estimated based on the decreased number of medical or surgical procedures performed during the pandemic and assuming the potential time needed to reschedule cancelled procedures after lockdown [9, 18]. However, relatively few studies have attempted to estimate the actual delay experienced by individual patients. The compensation effects of system adaptation on resource capacity (e.g., adjustment of treatment regimen/protocols) by oncologists might have resulted in more personalized treatments without lengthening their delays during the COVID-19 pandemic, as shown in several cancer types [19, 20]. To date, real-time data on this aspect is still scarce. A standardized approach for measuring the time interval to treatment to better estimate its impact on patient outcomes routinely, as well as under pandemic contingency, is of outmost importance [3, 16, 21].

This study aims to investigate the extent to which the COVID-19 pandemic modified the delay to first treatment and surgery among newly diagnosed patients.

## Materials and methods

We used data from the PAPESCO-19 (PAtients et PErsonnels de Santé des Centres de Lutte Contre le Cancer pendant l’épidémie de COVID-19), a multicenter cohort study that took place in 4 French Comprehensive Cancer Centers, located in Angers, Clermont-Ferrand, Nancy, and Nantes, as previously reported [22, 23]. Participants were eligible irrespective of whether they had COVID-19 symptoms or not. Between June 17, 2020 and Jun 11, 2021, 1,233 cancer patients and 1,071 healthcare workers were recruited and followed-up for one year. Patients were either under surveillance or undergoing active treatments. Of note, as a non-interventional study the PAPESCO-19 protocol did not modify their usual treatment. This study was registered at ClinicalTrials.gov, Identifier: NCT04421625.

All participants signed a written consent form, and the study was conducted in accordance with the Declaration of Helsinki. The Ethics Committee (CPP-IDF VIII, Boulogne-Billancourt) approved our study number 20.04.15 on May 15, 2020.

### Study population

In the present analysis, the subpopulation of the PAPESCO-19 study consisted of patients with localized BC which was diagnosed in one of the four cancer centers mentioned above; we included only patients with ongoing therapies, i.e., excluding those under surveillance (S1 Fig) Neoadjuvant chemotherapy (NACT) was prescribed according to national guidelines under the following conditions: ≥T2 or ≥N1 in HER2-positive or triple negative BC; hormone receptor-positive BC was treated with upfront surgery or NACT if conservative surgery was not possible upfront.

### Study outcomes

**Fig 1:**
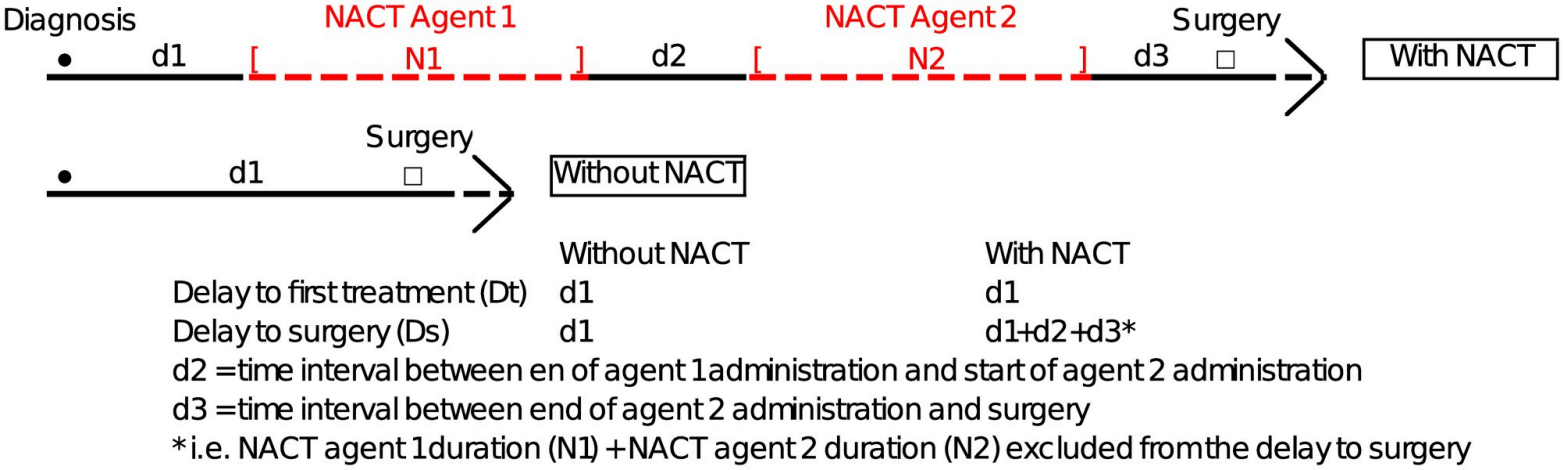
Presentation of study outcomes. NACT: Neo-adjuvant chemotherapy. For agents 1 and 2, see S4 Table for treatment protocol.

We measured the delay to first treatment (**Dt**) as the number of days between diagnosis and the first treatment. This delay is named **d1** regardless of whether the first treatment was NACT or surgery. We estimated the delay to surgery (**Ds**) as the time from diagnosis to surgery [16]. For patients without NACT, **Ds** equalled **d1** (Fig 1). For patients with NACT, it equalled **d1+d2+d3** (i.e., excluding NACT duration: N1+ N2 from the delay to surgery, as we focused only on lag time without ongoing treatment), as shown in the red dashed line in Fig 1.

### Covariables

We estimated pandemic severity using the rates of COVID-19 hospitalizations /100,000 inhabitants on a given date, from the county where the cancer centers are located (named **Ser** in what follows). Rates were retrieved from Santé publique France, the French national public health agency’s open data [24].

We also considered the two national lockdown periods in France in 2020: the first from March 17 to May 11, and the second from October 29 to December 12. We created a score variable to indicate the restriction level (**Sol**) scoring from 0 to 1 that classifies the periods as follows:

- **Sol** = 0, for the period without restriction measures (i.e., prior to 1^st^ national lockdown)
- **Sol** = 1 for the 1^st^ and 2^nd^ lockdown periods
- **Sol** = 0.5, for periods with moderate restriction measures (i.e., between the two lockdowns and after the 2^nd^ lockdown) [12, 25].

Finally, for each patient, we created a **composite pandemic index**, by multiplying mean **Ser** and mean **Sol** estimated for each patient’s treatment period.

We collected information on patient comorbidities including diabetes, hypertension, chronic renal failure, respiratory insufficiency, heart failure, and autoimmune diseases. We categorized patients with having at least one comorbidity (n ≥1) or no comorbidities (n = 0). We dichotomized age at inclusion, ≤55 and >55 years, based on the study population’s median age (Table 1). For the composite pandemic index we set the cut-off value at 50, as defined by the ATIH National Agency for Hospitalisation Information, for reporting for COVID-19 hospitalization rates in 2020 [26].

**Table 1:** Population characteristics (N = 187).

Characteristics	N	(%)
Age at inclusion		
Median (IQR)	55 (45–66)	
18–49	67	(36)
50–64	65	(35)
65–74	43	(23)
≥75	12	(6)
Center of inclusion		
Nantes	34	(18)
Angers	66	(35)
Clermont-Ferrand	36	(19)
Nancy	51	(27)
Diagnosis		
Before Lockdown	99	(53)
During 1st Lockdown	23	(12)
After 1st, before 2nd Lockdown	51	(27)
During 2nd Lockdown	4	(2)
After 2nd Lockdown	10	(5)
Surgery		
Before Lockdown	46	(25)
During 1st Lockdown	27	(14)
After 1st, before 2nd Lockdown	72	(39)
During 2nd Lockdown	11	(6)
After 2nd Lockdown	31	(17)
N of comorbidities		
0	127	(68)
1 or more	60	(32)
Comorbidities*		
Diabetes	13	(7)
Hypertension	37	(20)
Renal insufficiency	4	(2)
Respiratory insufficiency	9	(5)
Heart failure	6	(3)
Autoimmune diseases	5	(3)

*Patients may have more than one comorbidity

### Statistical analyses

We used descriptive statistics to present patient, treatment, and pandemic characteristics. We reported mean standard deviation (SD) or median and interquartile range (IQR, i.e., 25^th^ and 75^th^ percentiles), depending on the distributions. We performed Wilcoxon rank-sum to test whether there was a difference in age and in the proportion of patients with at least one comorbidity between patients diagnosed before and after the start of the first lockdown.

We used a swimmer-plot to visually examine the impact of the pandemic on times to treatment. Each swim lane represents an individual patient’s experience from diagnosis to surgery over time. This plot includes the period during which patients received NACT and the period during which there were no ongoing therapies. It also provides information on the first and second French national lockdowns and the inclusion period. We sorted patients by “with” and “without” NACT and the date of diagnosis.

We performed a generalized linear model (GLM) with a log link function to fit the distribution characteristics of the outcome, and a gamma distribution to optimize the errors to approximate normal distribution.

In univariable analysis, we tested the associations between outcomes, and single covariables. We compared the delays in patients diagnosed before and after the start of the first lockdown, and similarly in patients having surgery.

In the multivariable analysis, we ran three models. In the first model, we chose time to first treatment as the outcome. We used the composite pandemic index as the covariable indicating the pandemic situation, rather than the criteria of being diagnosed before or after lockdown. In the second and the third models, we used the delay to surgery as the outcome, separately in patients with and without NACT.

Additionally, we performed a sensitivity analysis by dichotomizing the time to first treatment with a cut-off of 60 days [16] and used logistic regression with the same covariables as described above.

## Results

**Fig 2:**
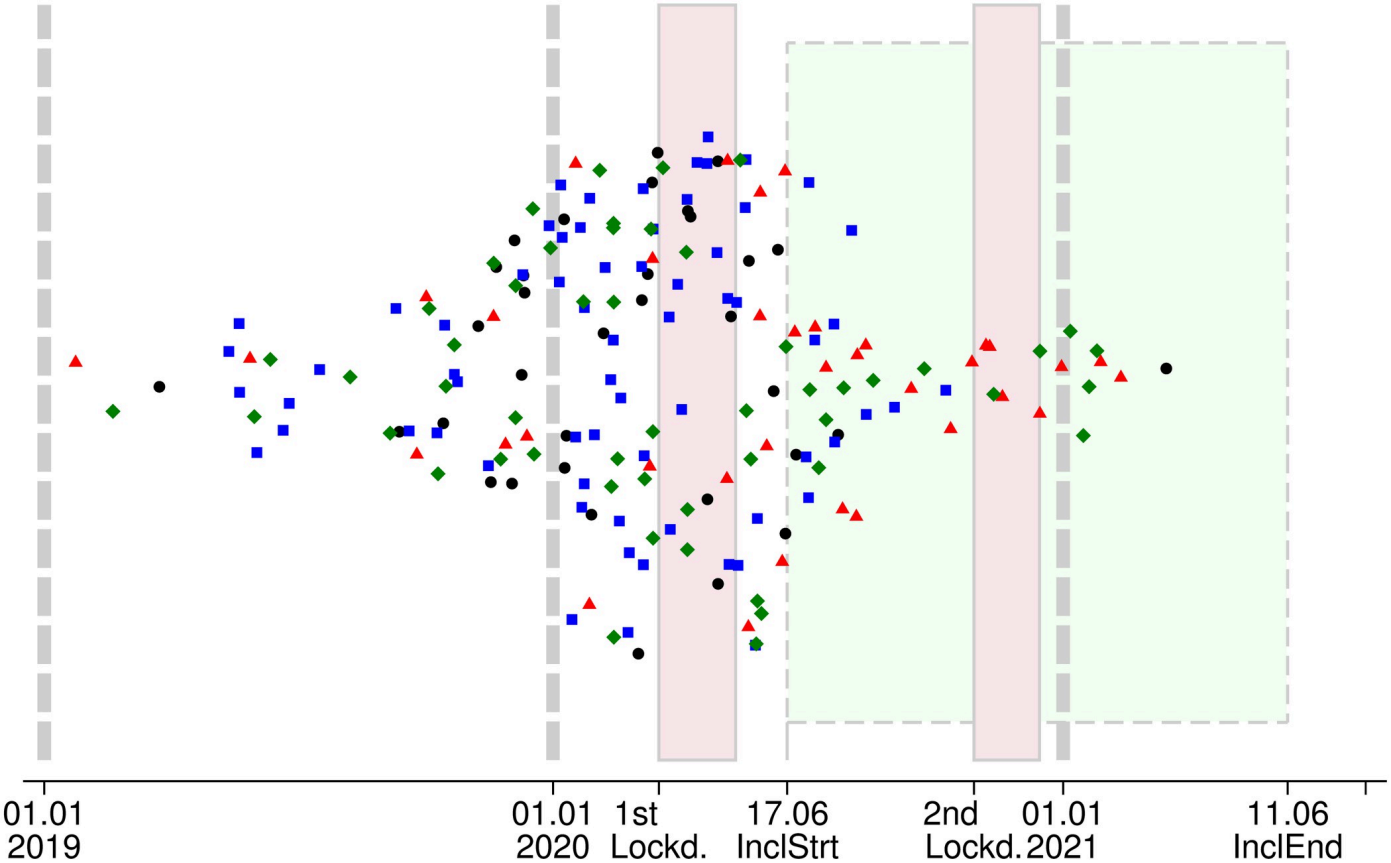
Diagnosis timeline. Date of diagnosis of localized breast cancer patients (N = 187) included in the PAPESCO-19 prospective study. Lockd.–Lockdown, InclStrt–start of study inclusion, InclEnd–end of study inclusion; Cranberry zones with solid contour– 1^st^ and 2^nd^ French national Lockdowns (from March 17 to May 11, 2020 and from October 30 to December 15, 2020, respectively). Lime zone delimited by dashes: inclusion period of the PAPESCO-19 study. Vertical dash lines: the first day of 2019, 2020 and 2021. Black circle: Nantes, Blue square: Angers; Red triangle: Clermont-Ferrand; Green diamond: Nancy; Vertical jitter has been added to reduce scatters from overlapping.

The study population included 187 women with localized BC underwent surgery in the Nantes, Angers, Clermont-Ferrand, and Nancy centers, as reported in the flowchart (S1 Fig). The diagnosis date ranged from January 23, 2019 to March 16, 2021 (Fig 2). Forty-six patients (25%) received NACT and surgery before the COVID-19 period, and 141 patients (75%) received all or part of their treatment during the COVID-19 period (Table 1).

Fifty-three percent (N = 99/187) of the women were diagnosed before the first lockdown, 14% (N = 27/187) during the two lockdowns (full restriction period), and 32% (N = 61/187) were diagnosed during moderate restriction periods (Table 1). Most patients were aged 50 and above. Within the study population, 41% (N = 77/187) underwent NACT. Age and the proportion of patients with at least one comorbidity were comparable between those diagnosed before and after the start of the first lockdown.

**Fig 3:**
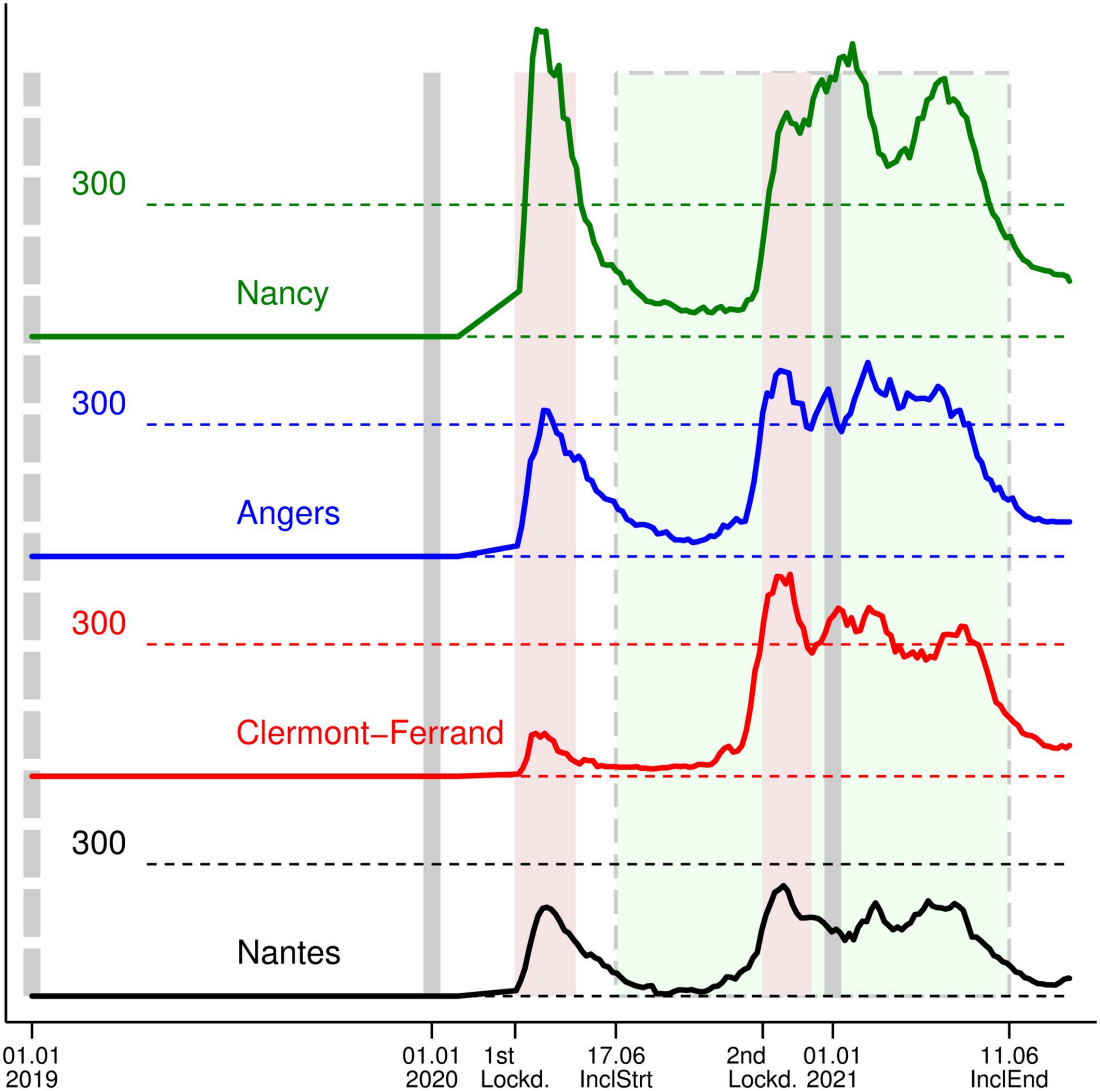
Ser (Number of hospitalized COVID-19 patients/100 000 inhabitants) at a given date in the county of the cancer centers of inclusion. Lockd.–Lockdown, InclStrt–start of study inclusion, InclEnd–end of study inclusion; Cranberry zones with solid contour– 1^st^ and 2^nd^ French national Lockdowns (from March 17 to May 11, 2020 and from October 30 to December 15 2020, respectively). Lime zone delimited by dashes: inclusion period of the PAPESCO-19 study. Vertical dash lines: the first day of 2019, 2020 and 2021. Black line: Nantes, Blue line: Angers; Red line: Clermont-Ferrand; Green line: Nancy; Vertical lines: first day of 2019, 2020, and 2021. The first COVID-19 case in France was reported on January 24, 2020. We assumed that the COVID-19 hospitalization rate was zero beforehand and a smooth linear increase between this date and the first date of data availability, which is March 18, 2020. Data source: Santé publique France—National Public Health Agency [24].

Ser (hospitalizations for COVID-19 per 100,000 inhabitants) during the 1st lockdown in Nancy’s cancer center, located in the most affected county, peaked at 695, compared to 201, 332, and 97 for the Nantes, Angers, and Clermont-Ferrand cancer centers, respectively. The county in which Clermont-Ferrand’s center is located experienced the worst situation during the 2nd lockdown, with Ser peaking at 454 (Fig 3), while it was 251, 423, and 441 for the Nantes, Angers and Nancy centers, respectively.

The composite pandemic index ranged from 0 to 574.1, with 142 distinct values for 187 patients. Overall, patients from the Nantes and Clermont-Ferrand cancer centers had the lowest index (median 18.6 and 13.6, IQR 0–44 and 10–120, respectively) followed by the Angers cancer center (median 43, IQR 0–92). Patients from the Nancy cancer center had the highest index (median 70.9, IQR 0–573) (Table 2).

**Table 2:** Delays to first treatment (Dt) and to surgery (Ds) and pandemic measurements.

	Date of diagnosis			
	Before&After 1st lockd. start (All)	Before 1st lockd. start	After 1st lockd. start	
	N of days	N of days	N of days	p-value
Delay to first therapy				
W/ and w/o NACT* (All)	N = 187	N = 99	N = 88	
Mean (SD)	45.3 (21.78)	44.7 (18.31)	46 (25.21)	
Median (IRQ)	42 (32–54)	42 (32–54)	42.5 (33–54)	0.82**
Range	7–208	11–106	7–208	
Delay to surgery				
Patients w/o NACT	N = 110	N = 55	N = 55	
Mean (SD)	43.7 (20.2)	43.1 (19.91)	44.3 (20.65)	
Median (IRQ)	39.5 (30–53)	37 (31–51)	45 (29–54)	0.55**
Range	7–106	11–106	7–104	
Patients w/ NACT	N = 77	N = 44	N = 33	
Mean (SD)	74.3 (28.17)	72.3 (24.18)	77 (32.95)	
Median (IRQ)	69 (60–86)	65 (56–87)	70 (63–85)	0.59**
Range	24–232	24–124	39–232	
Composite Pandemic Indicator				
All patients	N = 187	N = 99	N = 88	
Mean (SD)	69.7 (93.07)	22.4 (34.49)	122.9 (108.5)	
Median (IRQ)	30.7 (0.1–101.0)	3.4 (0–28.6)	93.1 (33.3–177.2)	
Range	69.7–93.07	0–155.7	6.5–573.1	
Nantes center	N = 34	N = 21	N = 13	<0.001***
Mean (SD)	28 (37.5)	10.7 (15.59)	55.8 (45.9)	
Median (IRQ)	18.6 (0–44.3)	0 (0–18.8)	46.8 (19.9–78.7)	
Range	0–177.6	0–58.8	6.5–177.6	
Angers center	N = 66	N = 39	N = 27	
Mean (SD)	58.9 (65.68)	19.8 (28)	115.2 (63.69)	
Median (IRQ)	43.1 (0–91.62)	0.1 (0–42.5)	100.2 (76.7–139.1)	
Range	0–286.3	0–116.3	24.2–286.3	
Clermont-Ferrand center	N = 36	N = 11	N = 25	
Mean (SD)	68.6 (92.18)	7.4 (7.36)	95.5 (99.51)	
Median (IRQ)	13.6 (9.7–119.9)	6.9 (0–12.5)	32.7 (11.4–161.4)	
Range	0–298.8	0–20.7	9.5–298.8	
Nancy center	N = 51	N = 28	N = 23	
Mean (SD)	112.2 (128.67)	40.5 (49.66)	199.5 (141.78)	
Median (IRQ)	70.9 (0–155.7)	13.8 (0–77.0)	176.7 (91.9–303.3)	
Range	0–574.1	0–155	33–573.1	

*NACT–Neoadjuvant Chemotherapy;

**Wilcoxon rank-sum test comparing before and after the start of the 1^st^ lockdown for the delay to first treatment and the delay to surgery;

***Wilcoxon rank-sum test comparing the composite pandemic index between centers for patients diagnosed after the start of the 1^st^ lockdown (N = 88)

**Fig 4:**
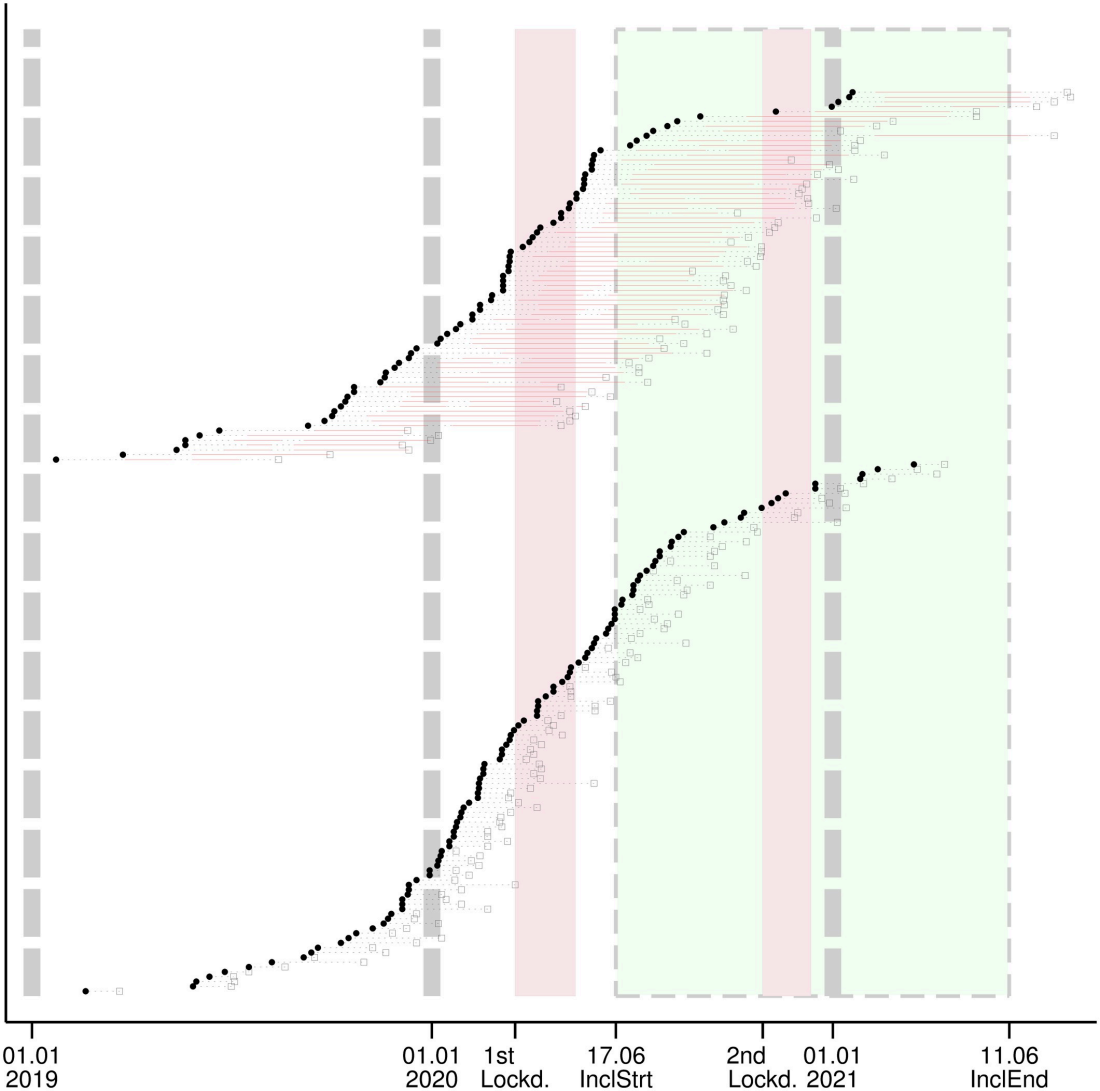
Swimmer plot from diagnosis to surgery and to first treatment. Patients grouped by: With (upper) and without (bottom) NACT. Black dots–diagnosis; Black squares–surgery; Horizontal dotted lines–delays between diagnosis, treatment and surgery; Horizontal red solid lines–NACT; Lockd.–Lockdown, InclStrt–start of study inclusion, InclEnd–end of study inclusion; Cranberry zones with solid contour– 1^st^ and 2^nd^ French national Lockdowns (from March 17 to May 11 2020 and from October 30 to December 15 2020, respectively). Lime zone delimited by dashes: inclusion period of the PAPESCO-19 study. Vertical dash lines: the first day of 2019, 2020 and 2021.

In this series, BC patients received their first treatment at a median of 42 (IQR: 32–54) days after diagnosis. No significant difference was observed in patients diagnosed before or after the start of the first lockdown (median 42, IQR 32–54 and median 42.5, IQR 33–54 days) (Table 2). There were no visual differences in delays in different pandemic periods, as shown in Fig 4.

Univariable analysis showed that patients aged over 55 years (p<0.05), and those included in Angers, Clermont-Ferrand and Nancy had longer delays to first treatment compared with Nantes (p<0.05). (S2 Table).

Multivariable analysis shows that the pandemic situation, represented here by the Composite Pandemic Index had only a small, non-significant, modifying effect on the time to first treatment and surgery. (Table 3). The fitted model predicted that the effect size of the highest pandemic index compared to the lowest index was -1.2 days. In contrast, the effect sizes of patient age (+5.6 days) and center of inclusion (+10 to +12.7 days) were larger than those of the pandemic index (S3 Table).

**Table 3:** Multivariable analysis.

	Outcome variable								
	Delay before 1st Trt.			Delay before surgery					
	All patients			Without NACT			With NACT		
	N = 186*			N = 110			N = 76		
	Coef.**	SE**		Coef.	SE		Coef.	SE	
Composite Pandemic Index (Ser x Sol)									
<50	REF		NS	REF		NS	REF		NS
> = 50	-0.089	0.065		-0.061	0.095		-0.022	0.07	
Age at inclusion									
< = 55 yrs	REF		<0.05	REF		NS	REF	0.061	NS
>55 yrs	0.131	0.064		0.122	0.1		0.156		
Center of inclusion									
Nantes	REF		<0.05	REF		NS	REF		NS
Angers	0.17	0.092		0.096	0.129		0.01	0.1	
Clermont-F.	0.3	0.099		0.283	0.135		0.232	0.11	
Nancy	0.24	0.095		0.289	0.124		-0.224	0.12	
N of comorbidities									
0	REF		NS	REF		NS	REF		NS
1 or more	0.006	0.071		0.059	0.104		0.049	0.07	
NACT***									
Without	REF		NS	-			-		
With	0.006	0.066		-			-		

* 1 outlier with extreme delay value has been excluded ;

** Coefficient and Standard Error;

*** NACT- Neoadjuvant Chemotherapy

In the sensitivity analysis, we found 15% of the patients who had a delay to first treatment of 60 days or more. This was not associated with any covariable.

## Discussion

In this study, we demonstrated that the COVID-19 pandemic did not change the delay to treatment in newly diagnosed patients with localized BC in four French comprehensive cancer centers. Our analysis showed that being diagnosed either before or after the start of the first lockdown, having a higher level of the composite pandemic index, did not modify the delay to first treatment and surgery.

Our study provides valuable insights into the real-world data analysis of delays in BC treatment during the COVID-19 pandemic. We used a direct comparison of individualized pandemic metrics, taking both pandemic severity and the level of lockdown restrictions into account, and the actual time to treatment observed. This enabled us to obtain an accurate measure of how pandemic severity and lockdown restrictions interacted to affect cancer treatment delays, estimating the differentiated impacts during lockdowns [25]. In France, during the second COVID-19 wave, despite its higher severity level compared to the first wave, restrictive measures set by the Government were progressively lightened, introducing more work flexibility and thus allowing more resources to support local hospital care [27]. Cancer care was more affected during the first wave, as opposed to the second wave, during which most activity related to cancer care was preserved [25]. Differentiating subperiods based on restriction measures helped us assess the potential impacts through the overall pandemic period. Conversely to our findings, in Italy, Vanni and colleagues observed a statistically significant longer time between breast biopsy and surgery in BC patients within the lockdown group (56 days) versus the pre-lockdown group (42 days) during the pandemic [28].

Our approach differs from that of previous studies in that they compared volumes of clinical and surgical procedures performed between the COVID-19 pandemic period and pre-pandemic period (e.g. 2015–2019) [9, 15, 18]. Such comparisons were likely to reflect not only the changes caused by the pandemic, but also the trends in BC treatment protocols that have resulted in changes in treatment delays over time, and might thus have overestimated the pandemic effect on delays [9, 15]. In light of the pandemic, BC care has a degree of flexibility that makes possible changes to treatment regimens and delivery settings [5, 18, 19, 29, 30]. Our analysis, carried out at the patient level, and taking into account heterogeneous regional pandemic stages and restriction policies that evolved over time had the advantage of providing our model with higher statistical power for detecting the actual effect of the pandemic on treatment delay.

Several reasons may explain why the COVID-19 pandemic did not affect time to treatment in our study population. In addition to the reduced number of newly diagnosed and treated cancer patients in 2020 in France (i.e., a 21% decrease between April-May 2020) [18], comprehensive cancer centers adapted treatment protocols, which reflects the resilience of the healthcare system [19, 30]. For example, outpatient treatments for metastatic BC were privileged during the pandemic, which ultimately reduced total admissions and might have compensated for the decrease in clinical procedures during the pandemic [29]. This helped preserve the capacity to treat patients with curative intent, particularly those requiring NACT or postoperative adjuvant chemotherapy. BC surgeries were limited to simple mastectomies, with reconstructive surgery being resumed once pandemic restrictions were lifted [5]. Finally, variations in the time to treatment were more strongly correlated with factors other than those pandemic-related, which dissimulated the effect of the pandemic [16]. Variations in delays stem from organisational or structural aspects of cancer treatment. Interestingly, the lowest frequency of BC therapy delays during the pandemic was found in specialized cancer centers, whereas, general hospitals were found to have much longer delays in a large European study [31]. This study observed substantial variations in treatment delays estimated in different studies, with percentages of patients affected by delays that varied from 3% to 76%, depending on therapy [30]. Additionally, addressing the issue of treatment delays associated with the COVID-19 pandemic is all the more challenging because there is no standardized definition of the time to treatment in cancer care, as a recent systematic review reports [3], although in the Netherlands guidelines recommend that “treatment (i.e., NACT, radiotherapy and/or surgery) is normally required within six weeks of the initial cancer diagnosis” [32]. The pre-pandemic times to treatment reported by oncologists in the study centers were within the same ranges as those we measured (S4 Table).

Despite the validity of our findings discussed above, we acknowledge some limitations. Our findings apply only to patients with localized BC. They should not be extrapolated to other cancers, which may have been more affected by the pandemic than BC [9, 15]. Furthermore, as the 4 cancer centers in our study did not have a specific COVID-19 unit, extrapolation of our results should be only made to cancer centers with the same setting. The time to treatment is likely to be influenced by factors unobserved in our analysis such as patients’ choice and changes in individuals’ health-seeking behavior. Social distancing measures concomitant to the lockdown measures might have affected patient-related access, delay to diagnosis and ultimately to treatment. Additionally, an important part of the cancer care continuum affected by the pandemic is likely to be the delay from symptom onset to diagnosis [3], which was not available in our analysis. It is worth noting that a 60-day delay from diagnosis to NACT or to surgery is associated with a significantly increased risk of breast cancer-specific mortality [16, 33, 34]. In our study, the same measurement had a 42-day median, before and after the start of the first French national lockdown, which was in line with existing guidelines [32]. Finally, in our sensitivity analysis, we did not find any significant factors associated with the delay of 60-days or more.

## Conclusion

Assessing the impact of the COVID-19 pandemic on delay to first treatment or surgery in newly diagnosed patients with localized BC is of particular importance. The study found evidence of no direct impact of the pandemic on the actual delay to treatment at the patient level. Our findings have the potential to help clinicians’ decision-making and BC care management. Our approach paves the way for future research to model a multiple component analysis of delay, including clinical protocols, patient preferences, and micro and macro-organization of care.

## Patient and public involvement

Patients and/or the public were not involved in the design, or conduct, or reporting, or dissemination plans of this research.

## Supporting information

S1 Fig Flowchart. (DOCX)

S2 Fig Histogram, distribution of composite pandemic index. (DOCX)

S1 Table Neoadjuvant therapy (NACT) protocol. (DOCX)

S2 Table Univariable analysis. (DOCX)

S3 Table Values predicted by the fitted model and effect sizes of covariables. (DOCX)

S4 Table Pre-pandemic delay to treatment in routine clinical care based on experts’ opinion from cancer centers. (DOCX)

## References

[1] Neal RD, Tharmanathan P, France B, Din NU, Cotton S, Fallon-Ferguson J, et al. Is increased time to diagnosis and treatment in symptomatic cancer associated with poorer outcomes? Systematic review. Br J Cancer. 2015 Mar 31;112 Suppl 1(Suppl 1):S92–107. doi: 10.1038/bjc.2015.48 25734382 PMC4385982

[2] Hanna TP, King WD, Thibodeau S, Jalink M, Paulin GA, Harvey-Jones E, et al. Mortality due to cancer treatment delay: systematic review and meta-analysis. BMJ. 2020 Nov 4;371:m4087. doi: 10.1136/bmj.m4087 33148535 PMC7610021

[3] Tope P, Farah E, Ali R, El-Zein M, Miller WH, Franco EL. The impact of lag time to cancer diagnosis and treatment on clinical outcomes prior to the COVID-19 pandemic: A scoping review of systematic reviews and meta-analyses. Harper DM, Boonstra P, editors. eLife. 2023 Jan 31;12:e81354. doi: 10.7554/eLife.81354 36718985 PMC9928418

[4] Abdel-Razeq H, Mansour A, Edaily S, Dayyat A. Delays in Initiating Anti-Cancer Therapy for Early-Stage Breast Cancer—How Slow Can We Go? Journal of Clinical Medicine. 2023;12(13). doi: 10.3390/jcm12134502 37445537 PMC10342560

[5] Budiarta M, Brennan M. The Impact of COVID-19 on Breast Cancer Treatment: A Systematic Review: Breast cancer treatment during pandemic. Arch Breast Cancer. 2022 Jul 30;9(4):421–38.

[6] Maringe C, Spicer J, Morris M, Purushotham A, Nolte E, Sullivan R, et al. The impact of the COVID-19 pandemic on cancer deaths due to delays in diagnosis in England, UK: a national, population-based, modelling study. The Lancet Oncology. 2020 Aug 1;21(8):1023–34. doi: 10.1016/S1470-2045(20)30388-0 32702310 PMC7417808

[7] Bosch X, Montori-Palacin E, Martínez-Ferrer R, Aldea A, Moreno P, López-Soto A. Time intervals in the care pathway to cancer diagnosis during the COVID-19 pandemic: A large retrospective study from a high-volume center. Int J Cancer. 2023 Feb 1;152(3):384–95. doi: 10.1002/ijc.34260 36053784 PMC9539134

[8] Han X, Yang NN, Nogueira L, Jiang C, Wagle NS, Zhao J, et al. Changes in cancer diagnoses and stage distribution during the first year of the COVID-19 pandemic in the USA: a cross-sectional nationwide assessment. The Lancet Oncology. 2023 Aug 1;24(8):855–67. doi: 10.1016/S1470-2045(23)00293-0 37541271

[9] Bardet A, Fraslin AM, Marghadi J, Borget I, Faron M, Honoré C, et al. Impact of COVID-19 on healthcare organisation and cancer outcomes. European Journal of Cancer. 2021 Aug 1;153:123–32. doi: 10.1016/j.ejca.2021.05.012 34153714 PMC8213441

[10] Sud A, Jones ME, Broggio J, Loveday C, Torr B, Garrett A, et al. Collateral damage: the impact on outcomes from cancer surgery of the COVID-19 pandemic. Annals of Oncology. 2020 Aug 1;31(8):1065–74. doi: 10.1016/j.annonc.2020.05.009 32442581 PMC7237184

[11] Glasbey J, Ademuyiwa A, Adisa A, AlAmeer E, Arnaud AP, Ayasra F, et al. Effect of COVID-19 pandemic lockdowns on planned cancer surgery for 15 tumour types in 61 countries: an international, prospective, cohort study. The Lancet Oncology. 2021 Nov 1;22(11):1507–17. doi: 10.1016/S1470-2045(21)00493-9 34624250 PMC8492020

[12] Bhangu A. Effect of COVID-19 pandemic lockdowns on planned cancer surgery for 15 tumour types in 61 countries: an international, prospective, cohort study. The Lancet Oncology. 2021 Nov 1;22(11):1507–17. doi: 10.1016/S1470-2045(21)00493-9 34624250 PMC8492020

[13] You B, Ravaud A, Canivet A, Ganem G, Giraud P, Guimbaud R, et al. The official French guidelines to protect patients with cancer against SARS-CoV-2 infection. Lancet Oncol. 2020 May;21(5):619–21. doi: 10.1016/S1470-2045(20)30204-7 32220659 PMC7118635

[14] Gligorov J, Bachelot T, Pierga JY, Antoine EC, Balleyguier C, Barranger E, et al. [COVID-19 and people followed for breast cancer: French guidelines for clinical practice of Nice-St Paul de Vence, in collaboration with the Collège Nationale des Gynécologues et Obstétriciens Français (CNGOF), the Société d’Imagerie de la Femme (SIFEM), the Société Française de Chirurgie Oncologique (SFCO), the Société Française de Sénologie et Pathologie Mammaire (SFSPM) and the French Breast Cancer Intergroup-UNICANCER (UCBG)]. Bull Cancer. 2020 May;107(5):528–37. 32278467 10.1016/j.bulcan.2020.03.008 PMC7118684

[15] Le Bihan-Benjamin C, Rocchi M, Putton M, Méric JB, Bousquet PJ. Estimation of Oncologic Surgery Case Volume Before and After the COVID-19 Pandemic in France. JAMA Netw Open. 2023 Jan 3;6(1):e2253204. doi: 10.1001/jamanetworkopen.2022.53204 36701152 PMC9880797

[16] Bleicher RJ, Ruth K, Sigurdson ER, Beck JR, Ross E, Wong YN, et al. Time to Surgery and Breast Cancer Survival in the United States. JAMA Oncol. 2016 Mar;2(3):330–9. doi: 10.1001/jamaoncol.2015.4508 26659430 PMC4788555

[17] Dietz JR, Moran MS, Isakoff SJ, Kurtzman SH, Willey SC, Burstein HJ, et al. Recommendations for prioritization, treatment, and triage of breast cancer patients during the COVID-19 pandemic. the COVID-19 pandemic breast cancer consortium. Breast Cancer Res Treat. 2020 Jun;181(3):487–97. doi: 10.1007/s10549-020-05644-z 32333293 PMC7181102

[18] Blay JY, Boucher S, Le Vu B, Cropet C, Chabaud S, Perol D, et al. Delayed care for patients with newly diagnosed cancer due to COVID-19 and estimated impact on cancer mortality in France. ESMO Open. 2021 Jun 1;6(3):100134. doi: 10.1016/j.esmoop.2021.100134 33984676 PMC8134718

[19] Tlemsani C, Arrondeau J, De Percin S, Gataa I, Bretagne M, Ajgal Z, et al. Impact of the COVID-19 pandemic on the management of cancer patients: the experience of the cancer outpatients department of a university hospital in Paris. Clin Med (Lond). 2021 Sep;21(5):e552–5. doi: 10.7861/clinmed.2020-0666 34341004 PMC8439500

[20] Lo Giudice G, Colella G, Boschetti CE, Colella C, Tartaro G, Cirillo N. Increased Delay in Diagnosis, but Not Treatment, Among Patients With Oral Cancer During the COVID-19 Pandemic. JAMA Otolaryngology–Head & Neck Surgery. 2023 Jan 1;149(1):91–2. doi: 10.1001/jamaoto.2022.3652 36394852 PMC9673025

[21] Luo Q, Steinberg J, O’Connell DL, Grogan PB, Canfell K, Feletto E. Changes in cancer incidence and mortality in Australia over the period 1996–2015. BMC Res Notes. 2020 Dec 10;13(1):561. doi: 10.1186/s13104-020-05395-6 33303018 PMC7726606

[22] Zhou K, Blanc-Lapierre A, Seegers V, Boisdron-Celle M, Bigot F, Bourdon M, et al. Anosmia but Not Ageusia as a COVID-19-Related Symptom among Cancer Patients—First Results from the PAPESCO-19 Cohort Study. Cancers. 2021;13(14). doi: 10.3390/cancers13143389 34298605 PMC8303411

[23] Seegers V, Rousseau G, Zhou K, Blanc-Lapierre A, Bigot F, Mahammedi H, et al. COVID-19 Vaccination Campaign in Cancer Patients and Healthcare Workers-Results from a French Prospective Multicenter Cohort (PAPESCO-19). Cancers. 2022;14(22). doi: 10.3390/cancers14225547 36428640 PMC9688516

[24] Santé publique France. Données hospitalières relatives à l’épidémie de COVID-19 (SIVIC) [Internet]. 2023 [cited 2023 Jun 11]. data.gouv.fr/organizations/sante-publique-France.

[25] Le Bihan Benjamin C, Simonnet JA, Rocchi M, Khati I, Ménard E, Houas-Bernat E, et al. Monitoring the impact of COVID-19 in France on cancer care: a differentiated impact. Scientific Reports. 2022 Mar 10;12(1):4207. doi: 10.1038/s41598-022-07984-w 35273304 PMC8908298

[26] French agency for hospitalisation information (ATIH—Agence technique de l’information sur l’hospitalisaion). Overview—COVID-19 inpatient admissions in 2020 [Synthèse—Les prises en charge hospitalières de la COVID-19 en 2020] [Internet]. 2021. https://www.atih.sante.fr/sites/default/files/public/content/4144/aah_2020_analyse_covid.pdf.

[27] Or Z, Gandré C, Durand Zaleski I, Steffen M. France’s response to the Covid-19 pandemic: between a rock and a hard place. Health Econ Policy Law. 2022 Jan;17(1):14–26. doi: 10.1017/S1744133121000165 33662232 PMC8007943

[28] Vanni G, Tazzioli G, Pellicciaro M, Materazzo M, Paolo O, Cattadori F, et al. Delay in Breast Cancer Treatments During the First COVID-19 Lockdown. A Multicentric Analysis of 432 Patients. Anticancer Res. 2020 Dec;40(12):7119–25. doi: 10.21873/anticanres.14741 33288611

[29] Cardoso F, Kyriakides S, Ohno S, Penault-Llorca F, Poortmans P, Rubio IT, et al. Early breast cancer: ESMO Clinical Practice Guidelines for diagnosis, treatment and follow-up†. Ann Oncol. 2019 Aug 1;30(8):1194–220. doi: 10.1093/annonc/mdz173 31161190

[30] Morgan D, James C. Ready for the Next Crisis? Investing in Health System Resilience [Internet]. Organisation for Economic Co-operation and Development. 2023. 475 p. Available from: https://www.oecd-ilibrary.org/content/component/648e8704-en.

[31] Gremke N, Griewing S, Bausch E, Alymova S, Wagner U, Kostev K, et al. Therapy delay due to COVID-19 pandemic among European women with breast cancer: prevalence and associated factors. J Cancer Res Clin Oncol. 2023 Oct;149(13):11749–57. doi: 10.1007/s00432-023-05065-7 37405476 PMC10465653

[32] Filipe MD, van Deukeren D, Kip M, Doeksen A, Pronk A, Verheijen PM, et al. Effect of the COVID-19 Pandemic on Surgical Breast Cancer Care in the Netherlands: A Multicenter Retrospective Cohort Study. Clin Breast Cancer. 2020 Dec;20(6):454–61. doi: 10.1016/j.clbc.2020.08.002 32888855 PMC7413119

[33] de Melo AC, Thuler LCS, da Silva JL, de Albuquerque LZ, Pecego AC, Rodrigues L de OR, et al. Cancer inpatients with COVID-19: A report from the Brazilian National Cancer Institute. PLoS One. 2020;15(10):e0241261. doi: 10.1371/journal.pone.0241261 33104715 PMC7588058

[34] McLaughlin JM, Anderson RT, Ferketich AK, Seiber EE, Balkrishnan R, Paskett ED. Effect on survival of longer intervals between confirmed diagnosis and treatment initiation among low-income women with breast cancer. J Clin Oncol. 2012 Dec 20;30(36):4493–500. doi: 10.1200/JCO.2012.39.7695 23169521 PMC3518728

