# Supplementary material for: Did the COVID-19 pandemic delay treatment for localized breast cancer patients? A multicenter study
Source: PLoS One. 2024 May 31;19(5):e0304556. doi: 10.1371/journal.pone.0304556 (PMC11142554; doi:10.1371/journal.pone.0304556)
Supplement: S1 Fig — (DOCX) [file pone.0304556.s001.docx]

Did the COVID-19 pandemic delay treatment for localized breast cancer patients? A multicenter study

Supporting Materials

**S1 Fig. Flowchart**
