# Supplementary material for: Did the COVID-19 pandemic delay treatment for localized breast cancer patients? A multicenter study
Source: PLoS One. 2024 May 31;19(5):e0304556. doi: 10.1371/journal.pone.0304556 (PMC11142554; doi:10.1371/journal.pone.0304556)
Supplement: S2 Fig — (DOCX) [file pone.0304556.s002.docx]

Did the COVID-19 pandemic delay treatment for localized breast cancer patients? A multicenter study

Supporting Materials

**S2 Fig. Histogram, distribution of composite pandemic index**

| A) All centres N=187  | B) Centre Nantes N=34  |
| --- | --- |
| C) Angers Centre N=66 | D) Clermont-Ferrand Centre N=36  |
| E) Nancy Centre N=51   |  |
