# Supplementary material for: Did the COVID-19 pandemic delay treatment for localized breast cancer patients? A multicenter study
Source: PLoS One. 2024 May 31;19(5):e0304556. doi: 10.1371/journal.pone.0304556 (PMC11142554; doi:10.1371/journal.pone.0304556)
Supplement: S1 Table — (DOCX) [file pone.0304556.s003.docx]

Did the COVID-19 pandemic delay treatment for localized breast cancer patients? A multicenter study

Supporting Materials

**S1 Table. Neoadjuvant therapy (NACT) protocol.**

| 1. | 3EC100/3 weeks followed by 12 weekly Paclitaxel or 3 Docetaxel/3 weeks |
| --- | --- |
| 2. | 3EC100/3weeks followed by 12 weekly Paclitaxel + trastuzumab or 3 Docetaxel + trastuzumab/3 weeks followed by trastuzumab or trastuzumab emtansine/3 weeks for 13 injections |
| 3. | 4AC/2 weeks followed by 12 weekly Paclitaxel |

AC-Adriamycine Cyclophosphamide; EC-Epirubicine Cyclophosphamide. The main protocols in the neoadjuvant setting at the time of the COVID-19 pandemic did not include immunotherapy
