# Supplementary material for: Did the COVID-19 pandemic delay treatment for localized breast cancer patients? A multicenter study
Source: PLoS One. 2024 May 31;19(5):e0304556. doi: 10.1371/journal.pone.0304556 (PMC11142554; doi:10.1371/journal.pone.0304556)
Supplement: S2 Table — (DOCX) [file pone.0304556.s004.docx]

Did the COVID-19 pandemic delay treatment for localized breast cancer patients? A multicenter study

Supporting Materials

**S2 Table. Univariable analysis**

| Patient characteristics | Outcome variable | | |
| --- | --- | --- | --- |
|  | Delay to 1st treatment | Delay to surgery | |
|  | All patients | w/o NACT | w/ NACT |
|  | N=186* | N=110 | N=76* |
|  | Coef/Sign | Coef/Sign | Coef/Sign |
| Age at inclusion | *<0.05* | NS | NS |
| <=55 yrs | REF | REF | REF |
| >55 yrs | 0.13 | 0.17 | 0.13 |
| Center of inclusion | *<0.05* | <0.05 | <0.001 |
| Nantes | REF | REF | REF |
| Angers | 0.18 | 0.15 | -0.01 |
| Clermont-Ferrand | 0.29 | 0.3 | 0.21 |
| Nancy | 0.27 | 0.32 | -0.23 |
| Diagnosis | *NS* | NS | NS |
| Before Lockdown | REF | REF | REF |
| After 1st Lockdown | -0.01 | 0.03 | -0.01 |
| Surgery | NS | NS | NS |
| Before Lockdown | REF | REF | -0.1 |
| After 1st Lockdown | 0.04 | 0.12 | -0.26 |
| N of comorbidities | NS | NS | NS |
| 0 | REF | REF | REF |
| 1 or more | 0.09 | 0.14 | 0.06 |
| Comorbidities |  |  |  |
| Diabetes | NS | NS | NS |
| without | REF | REF | REF |
| with | -0.03 | 0.05 | -0.02 |
| Hypertension | NS | NS | NS |
| without | REF | REF | REF |
| with | 0.09 | 0.15 | -0.04 |
| Renal insufficiency | NS | NS | NS |
| without | REF | REF | REF |
| with | 0.07 | -0.05 | 0.43 |
| Respiratory insufficiency | NS | NS | NS |
| without | REF | REF | REF |
| with | 0.07 | 0.09 | -0.01 |
| Heart failure | NS | NS | NS |
| without | REF | REF | REF |
| with | 0.07 | 0.15 | -0.11 |
| Autoimmune diseases | NS | NS | NS |
| without | REF | REF | REF |
| with | 0.09 | -0.01 | 0.29 |

* 1 outlier with extreme delay value has been excluded; NACT-Neoadjuvant Chemotherapy; Coef/Sign: Coefficient and significant level, NS: Non-significant
