# Supplementary material for: Did the COVID-19 pandemic delay treatment for localized breast cancer patients? A multicenter study
Source: PLoS One. 2024 May 31;19(5):e0304556. doi: 10.1371/journal.pone.0304556 (PMC11142554; doi:10.1371/journal.pone.0304556)
Supplement: S3 Table — (DOCX) [file pone.0304556.s005.docx]

Did the COVID-19 pandemic delay treatment for localized breast cancer patients? A multicenter study

Supporting Materials

**S3 Table. Values predicted by the fitted model and effect sizes of covariables**

|  | Outcome variable | | | | |  |
| --- | --- | --- | --- | --- | --- | --- |
|  | Delay to 1st Treatment |  | Delay to surgery | | |  |
|  | All patients |  | w/o NACT |  | w/ NACT |  |
|  | N=186* |  | N=110 |  | N=76* |  |
| Mean predicted value and effect size | Days | Diff. | Days | Diff. | Days | Diff. |
| Composite Pandemic Index (Ser x Sol) |  |  |  |  |  |  |
| <50 (Ref) | 45 | 1.2 | 43.7 | 0 | 75.5 | 5.7 |
| >=50 | 43.8 |  | 43.7 |  | 69.8 |  |
| Age at inclusion |  |  |  |  |  |  |
| <=55 yrs (Ref) | 41.5 | 5.6 | 39.6 | 7 | 67.8 | 9.8 |
| >55 yrs | 47.1 |  | 46.6 |  | 77.6 |  |
| Center of inclusion |  |  |  |  |  |  |
| Nantes (Ref) | 36.8 | - | 35.5 | - | 71.8 | - |
| Angers | 44.2 | 7.4 | 41.2 | 5.7 | 71.5 | 0.3 |
| Clermont-Ferrand | 49.6 | 13 | 47.7 | 12 | 90.2 | 18 |
| Nancy | 46.3 | 9.5 | 48.8 | 13 | 57.4 | -14 |
| N of comorbidities |  |  |  |  |  |  |
| 0 (Ref) | 43.2 | 4 | 43.2 | 4.7 | 43.2 | 2.2 |
| 1 or more | 47.2 |  | 47.9 |  | 45.4 |  |

* 1 outlier with extreme delay value has been excluded; Diff. – Mean differences; NACT-Neoadjuvant Chemotherapy

**S4 Table. Pre-pandemic delay to treatment in routine clinical care based on experts’ opinion from cancer centers**

| Patients with NACT |  |
| --- | --- |
| d1 between diagnosis and NACT start | 28-35 days |
| d2 between two NACT agents | 7-21 days |
| d3 between NACT end to surgery | 21-28 days |
| Patients without NACT |  |
| d1 between diagnosis and surgery | 35-42days |

NACT-Neoadjuvant Chemotherapy; d1, d2, d3: see also Fig.1 in main text
