# Supplementary material for: Did the COVID-19 pandemic delay treatment for localized breast cancer patients? A multicenter study
Source: PLoS One. 2024 May 31;19(5):e0304556. doi: 10.1371/journal.pone.0304556 (PMC11142554; doi:10.1371/journal.pone.0304556)
Supplement: S4 Table — (DOCX) [file pone.0304556.s006.docx]

Did the COVID-19 pandemic delay treatment for localized breast cancer patients? A multicenter study

Supporting Materials

**S4 Table. Pre-pandemic delay to treatment in routine clinical care based on experts’ opinion from cancer centers**

| Patients with NACT |  |
| --- | --- |
| d1 between diagnosis and NACT start | 28-35 days |
| d2 between two NACT agents | 7-21 days |
| d3 between NACT end to surgery | 21-28 days |
| Patients without NACT |  |
| d1 between diagnosis and surgery | 35-42days |

NACT-Neoadjuvant Chemotherapy; d1, d2, d3: see also Fig.1 in main text
